# Application of femoral nerve block combined with modified swelling anesthesia in high ligation and stripping of great saphenous vein

**DOI:** 10.3389/fsurg.2022.1086735

**Published:** 2023-01-06

**Authors:** Xiaobin Chen, Zhenwen Liu, Binbin Zhou, Zuyou Fan, Hu Zhao, Chen Lin

**Affiliations:** ^1^ Department of General Surgery, Fuzong Clinical Medical College, Fujian Medical University, Fuzhou, China; ^2^ Department of General Surgery, 900th Hospital of Joint Logistics Support Force; Fuzhou, China; ^3^ Department of General Surgery, Dongfang Hospital of Xiamen University, School of Medicine, Xiamen University, Fuzhou, China

**Keywords:** data collection and analysis great saphenous varices, femoral nerve block, tumescent anesthesia, curative effect, visual analogue scale

## Abstract

**Background:**

To analyze and explore the clinical efficacy of ultrasound guided femoral nerve block combined with modified swelling anesthetic solution in high ligation and stripping of the great saphenous vein.

**Methods:**

90 patients with varicose great saphenous vein of lower limbs undergoing high ligation and stripping of great saphenous vein were randomly divided into group A (femoral nerve block combined with modified swelling anesthesia), group B (simple swelling anesthesia) and group C (epidural anesthesia), with 30 patients in each group. The serum CRP level, operation duration, intraoperative blood loss, postoperative hospitalization time, total hospitalization cost, postoperative VAS score, preoperative and postoperative VCSS score, intraoperative mean arterial pressure and heart rate, postoperative related complications, and patients, satisfaction with diagnosis and treatment were compared among the three groups.

**Results:**

There was no significant difference in operation duration, intraoperative blood loss, postoperative complications, and preoperative and postoperative VCSS scores among the three groups (*P* > 0.05). The postoperative hospitalization time, postoperative VAS score and total hospitalization cost of patients in group A and B were lower than those in group C, and the postoperative hospitalization time and postoperative VAS score in group A were more significant (*P* < 0.05). Compared with group B, the fluctuation range of intraoperative mean arterial pressure and heart rate, and postoperative serum CRP level in group A and C were lower, especially in group A (*P* < 0.05). The three groups of patients were followed up regularly after surgery. The results showed that the number of postoperative complications in group A was lower than that in the other two groups (*P* < 0.05), and the postoperative complications of the three groups were effectively relieved after symptomatic treatment (dressing change, anti-infection, taking drugs to improve circulation, etc.). The satisfaction of patients in group A was significantly higher than that in groups B and C (*P* < 0.05).

**Conclusions:**

Ultrasound guided femoral nerve block combined with modified swelling anesthetic solution applied in high ligation and stripping of the great saphenous vein can significantly improve postoperative inflammatory stress reaction of patients, effectively ensure the safety and reliability of surgical progress, help to improve analgesia effect and accelerate physical rehabilitation, and has short hospitalization time, low medical cost, and high satisfaction of patients' diagnosis and treatment, which is worthy of widespread clinical promotion and reference.

## Introduction

Varicose great saphenous vein is a common vascular disease of the lower limb, which is mainly caused by venous valve insufficiency or abnormal increase in venous pressure of the lower limb. It is mainly manifested as asymptomatic tortuous expansion of lower limb veins, which may affect the appearance of the limbs in severe cases (1–3). In recent years, the incidence of varicose great saphenous veins has gradually increased, especially in women, and inadequate treatment may cause skin itching, pigmentation, ulceration of lower limbs, and even amputation in severe cases (4, 5). At present, the treatment of varicose great saphenous veins is mainly based on surgical intervention. High ligation and stripping of great saphenous vein has the characteristics of definite curative effect, simple operation and low recurrence rate, and is widely used in clinical practice (6). The surgical intervention is an invasive operation, which needs be carried out under the satisfactory anesthetic effect. At present, the anesthesia methods used for high ligation stripped of great saphenous vein mainly include the laryngeal mask anesthesia, epidural anesthesia, nerve block anesthesia, swelling anesthesia, etc. However, different anesthesia methods have different degrees of influence on the patient's surgical safety, hemodynamic fluctuations, and postoperative stress response (7–9). Therefore, choosing a more appropriate anesthesia method can effectively ensure the smooth operation, reduce the fluctuation of hemodynamics, reduce the stress reaction after operation, and improve the safety of operation. It is considered that nerve block anesthesia in the high ligation and stripping of the great saphenous vein can make the hemodynamic indexes of patients more stable, and at the same time, the incidence of postoperative complications is also lower, and swelling anesthetic solution can relieve postoperative pain and reduce bleeding in surgical operation (10). This study aims to analyze and explore the clinical effect of ultrasound-guided femoral nerve block combined with modified swelling anesthetic solution in high ligation and stripping of the great saphenous vein.

## Materials and methods

### Data collection

From January 2020 to January 2022, 90 patients who underwent high ligation and stripping of the great saphenous vein in the 900th Hospital of the Joint Logistic Support Force of the Chinese People's Liberation Army were selected. The patients were randomly divided into group A (femoral nerve block combined with modified swelling anesthesia), group B (simple swelling anesthesia) and group C (epidural anesthesia), with 30 cases in each group. Inclusion criteria: ① According to the Chinese Expert Consensus on the Diagnosis and Treatment of Chronic lower extremity Venous Diseases (11), primary varicose great saphenous vein was diagnosed by physical examination, color Doppler ultrasound or angiography. ② According to the clinical etiology anatomy pathophysiology (CEAP) classification of chronic venous diseases of lower extremities issued by the American Society of Phlebiology in 2004 (12), it was C2-C5. ③ All patients had surgical indications and underwent unilateral high ligation and exfoliation of the great saphenous vein. ④ Complete clinical data. Exclusion criteria: ① Patients with deep venous insufficiency (such as iliac and femoral vein occlusion, iliac vein stenosis, etc.). ② acute deep vein thrombosis of lower extremity. ③ combined with heart, lung, liver, kidney and other organ dysfunction. ④ Patients with coagulation dysfunction. ⑤ Patients with consciousness disorders and Intellectually Disabled. ⑥ Patients who refused to undergo regional swelling anesthesia. There was no significant difference in the demographics (age, BMI, gender, smoking and drinking) among the three groups (*P* > 0.05), as shown in Table 1. In addition, there was no significant difference in CEAP grading of varicose veins among the three groups (*P* > 0.05), as shown in Table 2. It indicates that the data from these 3 groups were comparable. All patients signed informed consent.

**Table 1:** Basic data of the three groups.

Group	N	Age (Year)	BMI (kg/m^2^)	Gender (Male/Female)	Smoking (Yes/No)	Drinking (Yes/No)
A	30	55.83 ± 13.81	23.57 ± 2.54	13/17	1/29	1/29
B	30	55.77 ± 13.14	24.70 ± 2.98	15/15	0/30	1/29
C	30	57.80 ± 15.29	24.50 ± 2.90	16/14	1/29	0/30
F/*χ*2	–	0.201	1.386	0.623	1.265	1.265
*P*	–	0.818	0.256	0.733	1.000	1.000

**Table 2:** Grade of varicose veins in three groups (*n*).

Group	N	C2	C3	C4a	C4b	C5	C6
A	30	13	5	6	4	0	2
B	30	10	3	9	6	1	1
C	30	9	1	4	8	2	6
χ2	–	1.261	2.848	2.450	1.653	1.886	4.461
*P*	–	0.622	0.284	0.323	0.488	0.770	0.136

### Preparation of modified swelling anaesthetic fluid and nerve blocker

The modified swelling anesthetic solution: 500 ml of normal saline, 20 ml of 2% ropivacaine, 200 mg of lidocaine injection, 0.25 mg of epinephrine and 10 ml of 5% sodium bicarbonate. Nerve blockers: 20 ml of normal saline, 20 ml of 2% ropivacaine and 200 mg of lidocaine injection.

### Therapeutic process

All patients were treated by high ligation and stripping of the great saphenous vein under different anesthesia methods. Group A: Femoral nerve block combined with modified swelling anesthesia. The patient was placed in the supine position, and an ultrasound probe was placed under the inguinal ligament after conventional disinfection and towel laying to obtain the position of the femoral nerve. Under the guidance of color ultrasound, the needle was inserted from the lateral thigh to the femoral nerve, and 2 ml–3 ml nerve blocker was injected after no blood was drawn back. After the nerve blocker was diffused to the femoral nerve, 10 ml nerve blocker was injected around the femoral nerve until the concentric circle phenomenon was shown under color Doppler ultrasound. Under the guidance of ultrasound, the modified swelling anesthetic solution was injected around the great saphenous vein along the main trunk and branches of the great saphenous vein until the great saphenous vein was fully collapsed. A longitudinal incision about 2 cm long was made 2 cm below the groin to free the great saphenous vein, which was severed 0.5 cm away from the root of the great saphenous vein and ligated at the root. Then, the venous dissector was delivered to the distal end to the middle of the leg, and a longitudinal incision of about 1 cm was made at the point of touching the dissector, the great saphenous vein was severed and ligated with the venous dissector. Finally, punctate stripping of local varicose veins was performed at the pre-marked branches of the great saphenous vein, and the communicating branches were ligated. The main trunk was stripped from the distal end to the proximal end, and the incision was sutured and wrapped with gauze eccentric compression. Group B: Simple swelling anesthesia. The patient was placed in a supine position, and after conventional disinfection and towel laying, the modified swelling anesthetic solution was injected around the great saphenous vein along the main trunk and branches of the great saphenous vein under the guidance of ultrasound until the great saphenous vein was fully collapsed. After the anesthesia took effect, the operation process was the same as that of group A. Group C: Epidural anesthesia. The patient was placed in the left lateral position, punctured at L_2∼3_ places, then injected with 3 ml of lidocaine and 10 ml of ropivacaine, and changed to the supine position after the anesthesia took effect. During the operation, the root of the great saphenous vein was dissociated and ligated, and then the blood was drained from the distal end to the proximal end with the esmarch tourniquet. The rest of the operation was the same as that of group A. All patients in the three groups were treated with elastic bandages for 14 days after operation, and wore grade 2 (23–32 mmHg) elastic socks for 6 months. The patients were disinfected and changed dressing every 3 days after operation, and the incision and pain were observed. Discharge criteria are no active bleeding in the operation area, pain numerical score ≤3, Homan sign negative in the affected limb, and free movement of the lower limbs. Six months after operation, the VCSS scores of three groups of patients were followed up in the three groups by SMS, telephone and outpatient.

### Observation target

General data such as age, gender, BMI, smoking history, drinking history, and CEAP grade of the patient at admission. Visual analogue scale (VAS) (13) was used to score the pain of patients in the three groups on the 1st, 2nd and 3rd day after surgery, with 0 points representing no pain and 10 points representing the most severe pain. The lower limb bandage was removed on the 3rd day after operation, and the skin ecchymosis of the thigh was observed. Venous clinical severity score (VCSS) (14) was used to score the venous condition of patients before treatment and 6 months after surgery. Serum CRP levels were detected before operation, on the 1st day after operation and on the 3rd day after operation. The operation duration, intraoperative blood loss, postoperative hospitalization time, total hospitalization cost and postoperative complications were compared among the three groups. Anesthesia safety observation indicators: Preoperative and intraoperative blood pressure and heart rate, and patients' satisfaction with the diagnosis and treatment (patients' subjective feelings scores for satisfaction with the diagnosis and treatment: very satisfied was 10 points, satisfied was 7–9 points, general was 4–6 points, dissatisfied was 1–3 points, and very dissatisfied was 0 points).

### Statistical analysis

SPSS 26.0 software was used for statistical analysis. Measurement data of normal distribution was expressed by mean ± standard deviation (x ± s), and independent sample t-test was used for comparison between groups. Counting data were expressed by *n* (%), and comparison between groups was performed by *χ*^2^ test or Fisher exact test (*n* < 5). Rank sum test was used to compare rank data. A two-sided test was used, and *P* < 0.05 was considered statistically significant.

## Results

### Comparison of surgical data

In terms of postoperative hospitalization time, the patients in groups A and B were lower than those in group C, and the effect of shortening hospitalization time in group A was more significant, and the difference was statistically significant (*P* < 0.05). In terms of total hospitalization expenses, the patients in group A and group B were lower than those in group C, among which group B was more significant in reducing hospitalization expenses, and the difference was statistically significant (*P* < 0.05). In terms of patient satisfaction, patients in groups A and B were higher than those in group C, among which patients in group A had higher satisfaction with diagnosis and treatment, and the difference was statistically significant (*P* < 0.05), as shown in Table 3.

**Table 3:** Comparison of surgery-related data among three groups (x ± s).

Group	Cases	Operation duration	Intraoperative blood loss	hospitalization time	Total hospitalisation costs	Satisfaction	Postoperative complications (Yes/No)
A	30	74.27 ± 27.91	21.00 ± 11.10	5.13 ± 1.22*	10,801.66 ± 3398.05*	8.93 ± 0.37*	1/29
B	30	75.67 ± 27.69	22.67 ± 9.44	5.77 ± 1.36*	8911.60 ± 1677.11*	7.87 ± 0.51*	3/27
C	30	76.27 ± 28.20	20.00 ± 7.54	9.03 ± 1.75*	12,661.42 ± 3563.20*	6.93 ± 1.08*	6/24
F/χ2	–	0.041	0.607	61.533	11.694	57.829	3.992
*P*	–	0.960	0.547	<0.001	<0.001	<0.001	0.146

* Note: Compared with group C, *P* < 0.05.

### Comparison of hemodynamic parameters

In terms of heart rate changes, there was no significant difference in preoperative heart rate among the three groups (*P* > 0.05), but there was a significant difference in operative center rate (*P* > 0.05). Among them, group B had the largest fluctuation range, followed by group A, and group C had the smallest fluctuation (*P* < 0.05). In terms of the change of mean arterial pressure, the fluctuation range of patients in group B was the largest, followed by that in group A, and that in group C was the smallest, with statistical significance (*P* < 0.05), as shown in Table 4.

**Table 4:** Comparison of hemodynamic parameters among three groups (x ± s).

Group	N	Heart rate		Mean arterial pressure	
		Preoperative	Intraoperative	Preoperative	Intraoperative
A	30	73.80 ± 5.99	70.17 ± 4.71*	116.73 ± 6.85	113.80 ± 6.83*
B	30	72.43 ± 4.86	80.23 ± 4.68*	118.10 ± 6.96	126.43 ± 5.59*
C	30	72.27 ± 5.379	71.27 ± 7.51*	117.73 ± 7.89	117.57 ± 11.07*
F	–	0.722	27.316	0.286	18.893
*P*	–	0.489	<0.001	0.752	<0.001

* Note: Compared with preoperative, *P* < 0.05.

### Comparison of postoperative pain

In terms of VAS score, on the 1st, 2nd and 3rd day after operation, the VAS scores of patients in group A and group B were lower than those in group C, and the effect of group A was more significant in reducing the VAS score after operation, with a statistically significant difference (*P* < 0.05), as shown in Table 5.

**Table 5:** Comparison of postoperative pain degree among three groups (x ± s).

Group	N	VCSS		VAS		
		Preoperative	6 months after surgery	Postoperative day 1	Postoperative day 2	Postoperative day 3
A	30	5.97 ± 0.41	1.07 ± 0.25	1.20 ± 0.41*	1.30 ± 0.47*	1.13 ± 0.35*
B	30	5.93 ± 0.52	1.17 ± 0.38	1.80 ± 0.71*	1.77 ± 0.77*	1.57 ± 0.86*
C	30	5.90 ± 0.40	1.03 ± 0.18	2.37 ± 1.22*	2.27 ± 0.98*	2.10 ± 0.96*
F	–	0.165	1.795	14.199	11.836	11.874
*P*	–	0.848	0.172	<0.001	<0.001	<0.001

* Note: Compared with group C, *P* < 0.05.

### Comparison of serum inflammatory response indexes

In terms of CRP level, on the 1st and 3rd day after operation, the CRP levels of patients in group A and C was lower than those of patients in group B, and among which the CRP level in group A was lower, with statistical significance (*P* < 0.05), as shown in Table 6.

**Table 6:** Comparison of CRP levels among three groups (x ± s).

Group	N	Preoperative	Postoperative day 2	Postoperative day 3
A	30	5.69 ± 0.65	7.93 ± 0.71*	5.80 ± 0.95*
B	30	6.02 ± 0.68	10.07 ± 0.73*	8.08 ± 0.82*
C	30	5.81 ± 0.74	8.89 ± 1.08*	6.67 ± 1.41*
F	–	1.707	47.007	33.614
*P*	–	0.187	<0.001	<0.001

* Note: Compared with group B, *P* < 0.05.

### Comparison of adverse reactions

Regular follow-up of patients in the 3 groups after operation showed that 1 case of superficial phlebitis occurred in group A after operation. There were 3 cases of postoperative complications in group B, including 1 case of superficial phlebitis, 1 case of mild edema of affected limb, and 1 case of soft tissue infection of lower limb. However, group C had the most postoperative complications (6 cases), including 3 cases of superficial phlebitis, 1 case of lower limb soft tissue infection, 1 case of stasis dermatitis and 1 case of incision infection. There was no significant difference in postoperative complications among the 3 groups (*P* < 0.05), as shown in Table 3. The postoperative complications of the three groups were effectively relieved after symptomatic treatment (dressing change, anti-infection, taking drugs to improve circulation, etc.).

## Discussion

Varicose veins are a kind of common chronic disease in clinic. Venous blood with blocked reflux compresses the vascular wall of the lower extremity, which further damages the function of venous valves, thereby causing a series of clinical complications and bringing great pain to people's lives (15). In terms of treatment, high ligation and stripping of the great saphenous vein is a traditional and commonly method for the treatment of great saphenous vein (16, 17). However, the traditional treatment also has certain defects, which are mainly reflected in the high incidence of postoperative complications (congestion, ecchymosis, superficial phlebitis, incision infection, etc.) (18, 19). In recent years, the related literature has proposed that different anesthesia methods combined with high ligation and stripping of the great saphenous vein in the treatment of great saphenous varices can achieve better clinical effect and effectively reduce the complications caused by the traditional operation (7, 9). At present, high ligation and stripping of the great saphenous vein is generally performed under epidural anesthesia. Although the curative effect is acceptable, epidural anesthesia may cause urinary retention, waist pain and discomfort, high hospitalization cost and long hospitalization time, which bring discomfort to patients. With the continuous development and improvement of minimally invasive technology, ultrasound-guided femoral nerve block anesthesia combined with other anesthesia in the surgical treatment of great saphenous varicose veins has been accepted by patients with fewer anesthetic side effects, high safety, minimally invasive aesthetics, faster postoperative recovery and better curative effect, which has high clinical popularization value (7, 9, 20, 21). Swelling anesthesia as a way of local anesthesia method, also known as the swelling technology, was first put forward by Klein (22) in 1987. In recent years, it has been widely applied in the surgical treatment, that is, by injecting the solution made up of epinephrine and lidocaine into the subcutaneous tissue, it makes the subcutaneous tissue edema, separates the tissue cell gap, and thus oppresses the tiny blood vessels, thus achieving the effects of local anesthesia to relieve pain, reduce bleeding and separate tissues. In this study, the traditional swelling anesthetic solution was modified by adding ropivacaine and sodium bicarbonate injection, combined with femoral nerve block in the high saphenous vein ligation and stripping, which greatly alleviated the tissue trauma during the operation and promoted the postoperative rehabilitation of patients, which was in line with the concept of fast track surgery.

The results of this study showed that the preoperative general conditions and degree of varicose veins were similar among the three groups, and there were no significant differences in operation duration, intraoperative blood loss, postoperative complications, preoperative and postoperative VCSS scores among the three groups (*P* > 0.05), suggesting that there is no significant difference in the medical safety and surgical treatment effect between the combined anesthesia group and the epidural anesthesia group and the simple swelling anesthesia group, and the application of ultrasound guided femoral nerve block combined with modified swelling anesthesia in high ligation and stripping of the great saphenous vein can achieve the same surgical safety and therapeutic effect. In terms of postoperative hospitalization time and VAS score on the 1st, 2nd and 3rd day after surgery, patients in combined anesthesia group and simple swelling anesthesia group were lower than those in epidural anesthesia group, suggesting that modified swelling anesthesia solution can significantly improve surgical trauma, reduce postoperative pain, and promote postoperative recovery of patients. The main reasons are considered as follows: The duration of ropivacaine and lidocaine was prolonged under the effect of adrenaline constriction of blood vessels, and the swelling effect combined with the modified swelling anesthetic solution not only reduced the stripping trauma caused by surgery, but also alleviated the inflammatory response caused by postoperative pain stress, so as to further reduce postoperative pain (23). In addition, the combined anesthesia group had a more significant effect in shortening the length of hospitalization time and reducing the postoperative VAS score, indicating that the combined anesthesia group had a better effect in improving surgical trauma and promoting postoperative recovery than the other two groups.

Ropivacaine can produce reversible block by blocking the inflow of sodium ions, thus achieving sedative and analgesic effects, and has the effect of stabilizing the hemodynamics of patients, and it also has synergistic effect when combined with local anesthetic drugs (24, 25). This study found that compared with the preoperative, the fluctuation range of heart rate and average arterial pressure in the simple swelling anesthesia group was the largest, followed by the combined anesthesia group, and the epidural anesthesia group is the smallest. It is suggested that the hemodynamic changes in the three groups are all changed during operation under different anesthesia methods, and the hemodynamic changes in the simple swelling anesthesia group are the largest. It is considered that the modified swelling anesthesia solution is injected around the great saphenous vein along the main trunk and branches of the great saphenous vein during operation, the pain stress reaction is strong, which affects the stability of the hemodynamics of patients to some extent. For the combined anesthesia group, ultrasound-guided femoral nerve block followed by modified swelling anesthesia can greatly reduce the intraoperative stress reaction of patients, effectively improve the stress state and stabilize hemodynamics.

C-reactive protein, also known as acute phase protein (CRP), is a kind of protein that rises sharply in the plasma when the body is infected or damaged, and it is closely related to inflammatory stress response (26). In this study, on the 1st and 3rd day after operation, the CRP levels of patients in the combined anesthesia group and the epidural anesthesia group were lower than that of patients in the simple swelling anesthesia group, and the CRP level in the combined anesthesia group was lower, indicating that the combined anesthesia group had a more significant effect in improving the surgical trauma of patients. The main reason is that the modified swelling anesthetic solution is injected around the great saphenous vein, effectively separating the tissue space around the blood vessels, and cooperating with the vasoconstriction caused by adrenaline, it can effectively compress the blind end of the collateral vessels and reduce postoperative subcutaneous tissue bleeding and ecchymosis. At the same time, the added sodium bicarbonate solution buffers the acidity of ropivacaine and lidocaine, reduces the tingling sensation caused by the local swelling anesthetic solution, reduces the inflammatory reaction, further reduces the postoperative pain, so that patients to get out of bed as soon as possible, reduce the occurrence of postoperative bed complications, shorten the length of postoperative hospitalization time, and then reduce the hospitalization cost. The results of this study showed that the total hospitalization cost of patients in combined anesthesia group and simple swelling anesthesia group were lower than that of patients in epidural anesthesia group, and the difference was statistically significant (*P* < 0.05). In addition, the patients' satisfaction with diagnosis and treatment in the combined anesthesia group and the simple swelling anesthesia group was higher than that of patients in the epidural anesthesia group, among which the patients' satisfaction of the combined anesthesia group was higher. Considering that compared with the epidural anesthesia group, the patients in the combined anesthesia group and the simple swelling anesthesia group do not need to fast water for 6 h before surgery, and they can eat immediately after surgery, and they do not need catheterization before surgery, which can avoid urethral injury and lumbar and back pain caused by epidural anesthesia, and reduce the cost of hospitalization, so patients' satisfaction with diagnosis and treatment is higher.

There are some limitations in this study. On the one hand, this study belongs to a retrospective study with a small sample and a single center; On the other hand, the follow-up time of this study is short and the types of complications involved were relatively limited. Therefore, in the later period, it is necessary to further expand the sample size for multi-center research, extend the follow-up time to verify the accuracy of the conclusion, and effectively apply it in clinic.

In conclusion, the femoral nerve block guided by color Doppler ultrasound in combination with modified fluid swelling anesthesia downlink great saphenous vein high ligation stripped, can significantly improve patients with postoperative inflammatory stress reaction, reduce the postoperative CRP levels, with a short length of hospital stay, lower health care costs, patient treatment satisfaction is high, effectively guarantee the safety and reliability of the operation progress at the same time, help to improve analgesia effect and speed up the body rehabilitation, which is worthy of clinical popularization and reference.

## Data Availability

The original contributions presented in the study are included in the article/Supplementary Material, further inquiries can be directed to the corresponding author/s.
